# Changes in productivity in healthcare services in the Kingdom of Saudi Arabia

**DOI:** 10.1186/s12962-022-00338-3

**Published:** 2022-01-25

**Authors:** Mohammed Khaled Al-Hanawi, Innocent F. Makuta

**Affiliations:** 1 Department of Health Services and Hospital Administration, Faculty of Economics and Administration, King Abdulaziz University, Jeddah, Saudi Arabia; 2 Health Economics Research Group, King Abdulaziz University, Jeddah, Saudi Arabia; 3 Department of Economics, University of Malawi, Zomba, Malawi

**Keywords:** Changes in technology, Healthcare services, Malmquist productivity index, Saudi Arabia, Scale efficiency, Technical efficiency, Total factor productivity

## Abstract

**Background:**

Amid the rising demand for healthcare services in Saudi Arabia, there is a need to monitor, evaluate, and improve performance in the delivery of these services. In this regard, the aim of this study was to estimate changes in total factor productivity (TFP) in healthcare services across the health system administrative regions in Saudi Arabia from 2006 to 2018. The contributions of changes in efficiency and technology to the observed changes in TFP were further evaluated.

**Methods:**

The data used for this study were extracted from annual Ministry of Health Statistical Yearbooks for the period of 2006–2018. TFP changes were estimated using the Malmquist Productivity Index, in which technology frontiers were constructed through data envelopment analysis. The changes in TFP were decomposed into changes in technology, changes in pure technical efficiency, and changes in scale efficiency following the Färe-Grosskopf-Norris-Zhang method. As robustness checks, we used bootstrapping to construct intervals and applied alternative decomposition methods. The changes in TFP and its sources were also compared between public and private hospitals.

**Results:**

Over the period from 2006 to 2018, TFP for healthcare services has decreased on average by 5.6% per year solely on account of a technical regress. Public hospitals registered a higher deterioration in productivity (6.0% per year) than private hospitals (4.8% per year).

**Conclusion:**

Using the available resources, there is potential for realising gains in productivity of healthcare services by addressing existing technological challenges. Since the decline in TFP is entirely a problem of technical regress, the primary solution should focus on strategies for achieving technical progress (innovation) through investment in more or better machinery, equipment, and structures for healthcare service provision.

## Introduction

There is an ever-increasing demand for healthcare services in the Kingdom of Saudi Arabia (KSA) due to a variety of factors, including a growing population, increased life expectancy, proliferation of lifestyle-related diseases, and the pursuit of universal health coverage [1]. This increased demand has to be satisfied with limited health resources. It is therefore imperative that the performance of hospitals be monitored, evaluated, and improved wherever possible so as to attain the maximum health outputs from the available health resources. Such improvements in performance have several benefits, including enhanced quality of services, increased number of patients served, and increased willingness of society to fund health services [2–5].

In order to monitor, evaluate, and improve performance in the delivery of healthcare services, it is necessary to measure such performance, track its evolution over time, and identify the sources of these changes. Unsurprisingly, there has been a proliferation of research aimed at these ends. Across the globe, such studies have measured performance either in terms of productivity or efficiency [6, 7]. However, in the KSA, almost all studies conducted to date on performance of the health sector have focused on measuring and/or comparing efficiency, demonstrating widespread inefficiencies among public hospitals [8, 9]. Further, these studies established that there has been little improvement in efficiency over time [10]. Some studies have further endeavoured to identify the determinants of the variations in efficiency levels within the healthcare system [9].

The current study departs from the aforementioned studies by focusing on productivity as a measure of performance in the delivery of healthcare services in the KSA. Productivity of a hospital (or healthcare system) represents the amount of healthcare outputs created from a given set of healthcare inputs. Over time, this productivity can change on two accounts: changes in efficiency and changes in technology [6, 11, 12]. The motivation for focusing on productivity and not only on efficiency, as was the case in the previous studies, is two-fold. Firstly, studies on efficiency implicitly assume that technology is fixed [12]; consequently, the contribution of technological change to performance is often ignored. The assumption that the level of technology is fixed, while tenable in a static analysis using cross-sectional data, is restrictive and hardly conforms to the real-world context where innovation over time is constantly changing production technologies. Secondly, by using cross-sectional data, most of the studies on efficiency have not assessed and/or compared changes in technical efficiency over time. Accordingly, these studies are unable to identify changes in efficiency over time and the sources of those changes. From a policy perspective, the limitations highlighted above may hinder the formulation and implementation of effective interventions. This is because interventions aimed at resolving efficiency issues are different from those aimed at technical progress/regress issues. That is, interventions must be customized to the problem to be effective.

Therefore, the aim of this study was to estimate productivity changes in the delivery of healthcare services that have occurred over time across the healthcare system administrative regions in the KSA, and to evaluate the contributions of the various components to those changes. To achieve this aim, the total factor productivity (TFP) changes over time were estimated. The estimated changes in TFP were decomposed into its components to better identify the sources of such changes: those related to efficiency and those related to technology. The changes in efficiency were then further decomposed into changes in pure technical efficiency and changes in scale efficiency. Comparisons of changes in TFP and its components were performed according to the ownership of hospitals (public vs private).

## Materials and methods

### Data and sources

Measurement of changes in productivity of a healthcare system involves a comparison of the amount of outputs produced by the healthcare system and the amount of inputs used to produce those outputs over time. The choice of inputs and outputs used in this study was guided both by prior research on the performance of hospitals and by availability of data. In the literature on hospital performance, the set of inputs typically includes the numbers of physicians, nurses, allied health professionals, and beds, along with operational expenses [9, 13, 14]. In some studies, a distinction was made between specialist and general physicians. The set of outputs generally includes the numbers of inpatient visits, outpatient visits, surgical operations, laboratory investigations, radiology films, and patients; bed turnover rate; bed occupancy rates; and average length of stay [7, 15]. Some studies adjusted for the case-mix and complexity of surgical procedures [14, 16, 17].

The number of inputs and outputs used in this study takes into account data constraints; the rule of thumb for studies on efficiency and productivity using data envelopment analysis (DEA) is that the number of decision-making units (DMUs) should be at least three times greater than the sum of the numbers of inputs and outputs [18]. With regards to inputs, we do not make any distinction between specialist and general physicians. Further, nurses, pharmacists, and all other allied health professionals are classified into a single ‘other health professionals’ group. By contrast, we restrict outputs to those that directly relate patient care, namely: the numbers of inpatient visits, outpatient visits, and surgical operations. We were unable to adjust for the complexity of surgical procedures given the nature of the data available. Therefore, a total of three inputs and three outputs were used in this analysis, which are defined in Table 1.

**Table 1:** Definitions of input and output variables

Variable	Definition
Inputs	
Number of physicians	Total number of physicians (specialists and general practitioners) employed in Saudi clinics and hospitals in a year
Number of other health professionals	Total number of nurses and allied health professionals (e.g. nurses, pharmacists, midwives, medical technicians, medical radiologists, physiotherapists) employed in Saudi clinics and hospitals in a year
Number of beds	Total number of hospital beds in a year
Outputs	
Number of outpatient visits	Total number of patients receiving outpatient treatment in hospitals within a year
Number of inpatient visits	Total number of patients receiving inpatient treatment within a year
Number of surgical operations	Total number of surgical operations in a year

All of the data used in this study were obtained from various editions of Saudi Arabia’s Ministry of Health (MOH) Statistical Yearbooks over a 12-year period from 2006 to 2018 [19]. Although the data provided in the Statistical Yearbooks are initially collected from hospitals, the data are aggregated and reported at the level of healthcare administrative regions, with a total of 20 regions. The number of public hospitals in the regions varies from 5 to 65, whereas that of private hospitals varies from 1 to 45. In each region, we treated public hospitals as separate DMUs from private hospitals because of the difference in the performance of these hospital types [20]. In some regions such as Bishah, Northern, Al-Jouf, Quarrayat, and Qunfudah, there are only outpatient clinics and no private hospitals. To reduce the heterogeneity of data, we excluded these outpatient clinics, as the scale and scope of services provided by these clinics are vastly different from those provided for inpatient hospital treatment [14]. Further, we only included DMUs with complete information for all variables over the study period in our analysis. DMUs with any missing information were excluded from the analysis to avoid bias in the estimated changes [21]. Therefore, even though our sample would potentially contain 40 DMUs (healthcare administrative regions), we used a total of 33 DMUs for which complete data were available for the full set of hospitals for the entire period under consideration. Data analysis was conducted in Stata version 16.1.

### Measuring changes in productivity

Given a set of *n* DMUs, each of which uses an *M* input vector *x* to produce an *S* output vector *y* in two periods (i.e. period *t* and period *t* + *1*), measuring changes in productivity involves two key tasks: (1) constructing the efficient frontier (also called the technology frontier) for each time period and (2) estimating the productivity index using the distance of the DMUs from the frontiers in each of these two periods.

The two most widely used approaches for construction of the efficient frontier are DEA and stochastic frontier analysis (SFA). Each of these approaches has its own merits and demerits [22, 23]. We adopted the non-parametric DEA approach for this study, since it allows for a multiple-input and multiple-output production process and does not make any assumption regarding the underlying production function [11, 12]. As noted by Hollingworth and Street [22], the information deficit regarding the production function is particularly acute in the production of healthcare, which is complex and individually tailored. The absence of the underlying production function makes DEA a more flexible approach than SFA [23].

The DEA-based efficient frontier is made up of the best-performing DMUs. Such a frontier can be constructed under two different assumptions regarding the returns to scale of the production technology, namely constant returns to scale (CRS) or variable returns to scale (VRS). We used both assumptions regarding technology in our analysis to provide a facility for decomposing the changes in TFP into its sources/components.

The output-oriented efficient frontiers were constructed following the approach of Banker et al. [24]. If each input and output is respectively given a weight of *u* and *v*, then the frontiers are constructed by solving the following linear programming problem:

1 $$\max z_{0} = \sum\limits_{r = 1}^{s} {u_{r} y_{r0} - u_{0} }$$

Subject to:

2a $$\sum\limits_{r = 1}^{s} {u_{r} y_{rj} - \sum\limits_{i = 1}^{m} {v_{i} x_{ij} - u_{0} } } \le 0$$

2b $$\sum\limits_{i = 1}^{m} {v_{i} x_{i0} } = 1$$

In this problem, ${u}_{0}$ can be negative, zero, or positive depending on whether the technology exhibits decreasing returns to scale, CRS, or increasing returns to scale. The second task involves estimating the productivity index using distances of the DMUs from the efficient frontiers in the two time periods. The distances were measured using distance functions, which we denote by *E*, that measure the distance from an actual DMU to the efficient frontier in an input–output space [14]. These distance functions can have an input orientation or an output orientation. The former orientation aims at minimisation of inputs for a given set of outputs, whereas the latter orientation aims at maximisation of the output for a given amount of inputs [12]. Arguments for using the input orientation include that hospitals have more control over inputs than outputs, whereas the motivation for using the output orientation may be that given a resource envelope, a hospital should produce as much output as possible [25, 26]. Regardless of the orientation adopted, the productivity index should increase if either input use decreases for the same level of output or output increases for the same level of input between two time periods [12]. Following the literature on the productivity of healthcare systems, we used the output-oriented distance function in this study [27, 28].

Changes in TFP were measured by the Malmquist Productivity Index (MPI), which was introduced by Caves et al. [29] to measure changes in productivity among DMUs based on the distance functions proposed by Malmquist [30]. In time periods *t* and *t* + *1*, the MPI is the ratio of the distance functions in the two periods, which can respectively be formulated as:

3 $$M^{t} = \frac{{E^{t} \left( {x^{t + 1} ,y^{t + 1} } \right)}}{{E^{t} \left( {x^{t} ,y^{t} } \right)}}$$

4 $$M^{t + 1} = \frac{{E^{t + 1} \left( {x^{t + 1} ,y^{t + 1} } \right)}}{{E^{t + 1} \left( {x^{t} ,y^{t} } \right)}}$$

Since the technologies in time periods *t* and *t* + *1* may differ, the general MPI for the two periods is calculated by taking the geometric mean of the MPI in Eqs. (3) and (4), yielding the following:

5 $$M = \left[ {\frac{{E^{t} \left( {x^{t + 1} ,y^{t + 1} } \right)}}{{E^{t} \left( {x^{t} ,y^{t} } \right)}}\frac{{E^{t + 1} \left( {x^{t + 1} ,y^{t + 1} } \right)}}{{E^{t + 1} \left( {x^{t} ,y^{t} } \right)}}} \right]^{\frac{1}{2}}$$

*M* > 1, *M* = 1, and *M* < 1 indicates growth, stagnation, and deterioration of productivity, respectively.

### Decomposing CHANGES in TFP

Productivity over time can vary due to changes in production technology, technical efficiency, or both [27]. The contribution of changes in technology and technical efficiency to the overall change in productivity can be estimated through decomposition. We decomposed the MPI using the Färe-Grosskopf-Norris-Zhang (FGNZ) method [11], in which the TFP in Eq. (5) can be decomposed into technical efficiency change (EFFCH) and technical/technological change (TECCH) as follows:

6 $$M = \frac{{E^{t + 1} \left( {x^{t + 1} ,y^{t + 1} } \right)}}{{E^{t} \left( {x^{t} ,y^{t} } \right)}}\left[ {\frac{{E^{t} \left( {x^{t + 1} ,y^{t + 1} } \right)}}{{E^{t} \left( {x^{t} ,y^{t} } \right)}}\frac{{E^{t + 1} \left( {x^{t + 1} ,y^{t + 1} } \right)}}{{E^{t + 1} \left( {x^{t} ,y^{t} } \right)}}} \right]^{\frac{1}{2}}$$

where the first term outside the square brackets captures EFFCH and the second term captures TECCH. TECCH shows the extent of changes in productivity on account of shifts in the efficient frontier, and thus reflects innovation (or lack thereof). A TECCH score of 1 means that the efficient frontier has not shifted between the two periods, indicating stagnation in production technology, whereas a score greater (less) than 1 means that the efficient frontier is shifting up (down) from one period to the other, indicating improvement (deterioration) in technology [31, 32]. By contrast, EFFCH measures the change in the distance of a DMU relative to the efficient frontiers in the two periods. Therefore, EFFCH reflects whether or not a DMU is catching up with its peers and is dependent on management practices. A score of 1 means that the DMU’s distance to the efficient frontier has not changed in the two periods, indicating stagnation in efficiency, whereas a score greater (less) than 1 means that a DMU is getting closer to (farther away from) the efficient frontiers, indicating improvement (deterioration) in efficiency [11].

By utilizing both CRS and VRS DEA frontiers, the change in technical efficiency (EFFCH) can further be decomposed into a pure efficiency change (PECH) component and a scale efficiency change (SECH) component. PECH measures the extent of change in efficiency of a DMU relative to the VRS frontier; a PECH score greater than 1 indicates gains in efficiency, whereas a score less than 1 implies reduction in efficiency [11, 32]. SECH measures the extent to which the distance from the scale-efficient point on the VRS frontier (relative to the CRS frontier) has changed. Thus, SECH measures the contribution to efficiency attributable to the production size of a DMU; a SECH score greater (less) than 1 indicates that a DMU has become more (less) scale-efficient [11, 32].

Computationally, SECH is given by:

7 $$SECH = \left[ {\frac{{{{E_{vrs}^{t} \left( {x^{t + 1} ,y^{t + 1} } \right)} \mathord{\left/ {\vphantom {{E_{vrs}^{t} \left( {x^{t + 1} ,y^{t + 1} } \right)} {E_{crs}^{t} \left( {x^{t + 1} ,y^{t + 1} } \right)}}} \right. \kern-\nulldelimiterspace} {E_{crs}^{t} \left( {x^{t + 1} ,y^{t + 1} } \right)}}}}{{{{E_{vrs}^{t} \left( {x^{t} ,y^{t} } \right)} \mathord{\left/ {\vphantom {{E_{vrs}^{t} \left( {x^{t} ,y^{t} } \right)} {E_{crs}^{t} \left( {x^{t} ,y^{t} } \right)}}} \right. \kern-\nulldelimiterspace} {E_{crs}^{t} \left( {x^{t} ,y^{t} } \right)}}}}\frac{{{{E_{vrs}^{t + 1} \left( {x^{t + 1} ,y^{t + 1} } \right)} \mathord{\left/ {\vphantom {{E_{vrs}^{t + 1} \left( {x^{t + 1} ,y^{t + 1} } \right)} {E_{crs}^{t + 1} \left( {x^{t + 1} ,y^{t + 1} } \right)}}} \right. \kern-\nulldelimiterspace} {E_{crs}^{t + 1} \left( {x^{t + 1} ,y^{t + 1} } \right)}}}}{{{{E_{vrs}^{t + 1} \left( {x^{t} ,y^{t} } \right)} \mathord{\left/ {\vphantom {{E_{vrs}^{t + 1} \left( {x^{t} ,y^{t} } \right)} {E_{crs}^{t + 1} \left( {x^{t} ,y^{t} } \right)}}} \right. \kern-\nulldelimiterspace} {E_{crs}^{t + 1} \left( {x^{t} ,y^{t} } \right)}}}}} \right]^{\frac{1}{2}}$$

and PECH is given by:

8 $$PECH = \frac{{E_{vrs}^{t + 1} \left( {x^{t + 1} ,y^{t + 1} } \right)}}{{E_{crs}^{t} \left( {x^{t} ,y^{t} } \right)}}$$

## Robustness checks

Robustness checks were performed with respect to the significance of observed changes and alternative decomposition methods. Firstly, to ascertain whether or not the observed changes in productivity and those of its components are significant from the benchmark score of one, we constructed confidence intervals using bootstrapping following the approach proposed by Simar and Wilson [33]. Secondly, there are debates in the literature regarding how the MPI should be decomposed [34]. As a robustness check with respect to decomposition, we also used the Ray-Desli (RD) decomposition method [35] and the Pastor-Lovell (PL) method. In contrast to the FGNZ and RD methods, which consider technologies in two adjacent periods being compared, the PL method constructs single global technology from all data for all units and all periods of the sample [34].

## Results

### Descriptive statistics

Table 2 presents the mean values for the input and output variables used in the study over the period 2006–2018. Since the study covered many years, for simplicity of presentation, the mean values are provided only at three-year intervals in the sample period. Column A of the table shows the overall means, while columns B through F show the means for particular years. From column B to column F, the pattern in the mean values of all variables is one of increase over time. By taking the ratio of 2018 values (column F) to 2006 values (column B), we obtain the proportionate growth in the variables over the period 2006–2018. On the input side, the numbers of physicians, other health professionals, and beds has grown by 141.7%, 140.3%, and 40.5%, respectively. On the output side, the numbers of outpatient visits, inpatient visits, and surgical operations has increased by 35.3%, 17.1%, and 63.3%, respectively. Evidently, while both inputs and outputs have expanded over time, inputs have grown by larger proportions than outputs in the study period.

**Table 2:** Summary statistics for input and output variables, 2006–2018

Variable	Overall (A)	2006 (B)	2009 (C)	2012 (D)	2015 (E)	2018 (F)
Number of physicians	1759	1053	1270	1735	2103	2545
Number of other health professionals	5541	2924	2586	4089	2238	7027
Number of beds	1555	1343	1361	1509	1750	1887
Number of outpatient visits	3,274,565	2,732,376	3,169,505	3,309,377	3,466,050	3,697,895
Number of inpatient visits	80,193	78,770	76,370	77,612	86,296	92,202
Number of surgical operations	24,643	19,057	22,530	24,627	27,841	31,123

### Changes in productivity and its sources

We computed annual average scores of changes in total factor productivity (TFPCH) using the MPI for hospitals in all healthcare administrative regions in the KSA from 2006 to 2018. Table 3 shows the average scores over the sample period determined by the FGNZ method. The average TFPCH index was 0.944, which implies TFP deterioration of 5.6% per year. Identical results were obtain using the RD method. The global method yielded an average TFPCH index of 0.951, which represents 4.9% annual erosion in productivity in health services.

**Table 3:** Changes in TFP and its components in the KSA, 2006–2018

MPI component	Method					
	FGNZ		RD		PL	
	Coefficient	95% CI	Coefficient	95% CI	Coefficient	95% CI
TFPCH	0.944	(0.9167, 0.9720)	0.944	(0.9164, 0.9723)	0.951	(0.9274, 0.9748)
EFFCH	0.989	(0.9661, 1.0114)	0.988	(0.9707, 1.0045)	0.989	(0.9662, 1.0113)
TECCH	0.955	(0.9285, 0.9817)	0.967	(0.9421, 0.9922)	0.962	(0.9369, 0.9869)

*MPI* Malmquist Productivity Index, *TFPCH* changes in total factor productivity, *TECCH* changes in technology, *EFFCH* changes in technical efficiency, *FGNZ* Färe-Grosskopf-Norris-Zhang, *RD* Ray-Desli, *PL* Pastor-Lovell, *CI* confidence interval

Next, we examined the contribution of changes in technology (TECCH) and technical efficiency (EFFCH), respectively, to changes in TFP. As shown in Table 3, all methods of decomposition yielded an average efficiency score that was statistically equivalent to 1, which implies that, on average, efficiency did not change over the sample period. The average technological change score ranged from 0.955 (by the FGNZ method) to 0.967 (by the RD method), with all scores being statistically lower than 1, indicating a technical regress between 3.7% and 4.5% per year. Therefore, the observed deterioration in TFP in healthcare services in the KSA was entirely due to technical regress.

We also analysed the changes in TFP and those of its components for public hospitals and private hospitals separately. As shown in Table 4, on the whole, public hospitals registered a deterioration in TFP between 4.2% and 6% per year (TFP scores ranging from 0.940 to 0.958). In the public sector, the deterioration in TFP resulted from technical regress alone with efficiency performance being constant. By contrast, the private hospitals exhibited stagnation in TFP and its components. This result should be interpreted with caution as the rule of thumb that the number of DMUs must be at least three times the sum of the number of inputs and outputs is violated.

**Table 4:** Changes in TFP and its components for public and private hospitals in the KSA, 2006–2018

MPI component	Method					
	FGNZ		RD		PL	
	Coefficient	95% CI	Coefficient	95% CI	Coefficient	95% CI
***Public hospitals***						
TFPCH	0.940	(0.9221, 0.9573)	0.940	(0.9224, 0.9570)	0.958	(0.9413, 0.9746)
EFFCH	0.980	(0.9516, 1.0080)	0.981	(0.9604, 1.0016)	0.983	(0.9606, 1.0050)
TECCH	0.959	(0.9331, 0.9850)	0.963	(0.9412, 0.9844)	0.975	(0.9529, 0.9966)
***Private hospitals***						
TFPCH	0.952	(0.8856, 1.0175)	0.953	(0.8859, 1.0172)	0.966	(0.9093, 1.0225)
EFFCH	1.003	(0.9647, 1.0407)	0.998	(0.9680, 1.0275)	1.003	(0.965, 1.0407)
TECCH	0.949	(0.8950, 1.0030)	0.976	(0.9161, 1.0350)	0.963	(0.9136, 1.0130)

*MPI* Malmquist Productivity Index, *TFPCH* changes in total factor productivity, *TECCH* changes in technology; *EFFCH* changes in technical efficiency, *FGNZ* Färe-Grosskopf-Norris-Zhang, *RD* Ray-Desli, *PL* Pastor-Lovell, *CI* confidence interval

Using the FGNZ method, we conducted a further decomposition of the changes in efficiency scores into pure efficiency change and scale efficiency change. As shown in Table 5, both components did not statistically deviate from a score of 1 when considering all hospitals taken together as well as when considering public and private hospitals separately.

**Table 5:** Changes in technical efficiency and its components in the KSA, 2006–2018

EFFCH component	Method					
	All hospitals		Public hospitals		Private hospitals	
	Coefficient	95% CI	Coefficient	95% CI	Coefficient	95% CI
PECH	0.988	(0.9703, 1.0049)	0.981	(0.9604, 1.0017)	0.998	(0.9681, 1.0275)
SECH	1.001	(0.9849, 1.0175)	0.999	(0.9770, 1.0205)	1.005	(0.9798, 1.0301)
EFFCH	0.989	(0.9661, 1.0114)	0.980	(0.9516, 1.0080)	1.003	(0.9647, 1.0407)

*PECH* changes in pure technical efficiency, *SECH* changes in scale efficiency, *EFFCH* changes in technical efficiency, *CI* confidence interval

## Discussion

In this study, we estimated changes in TFP in health services that have occurred in the KSA over time between 2006 and 2018 across the healthcare system administrative regions, and further evaluated the contribution of the various components to those changes. The results revealed that productivity of the healthcare system in the KSA has decreased by an average of 5.6% during this period. This result confirms the descriptive results showing that the growth in inputs has exceeded the growth in outputs over the study period. This reduction in TFP was wholly due to technical regress. That is, the deterioration of TFP in health services in the KSA is a technology problem. The observed technical regress could be attributed to either the low adoption of new technologies within the regions and/or to technical problems faced by using outdated technologies [28].

The annual deterioration of TFP by 5.6% implies that given the existing envelope of resources, it is possible to increase healthcare outputs by 5.6%. Since this deterioration in TFP is primarily accounted for by technical regress, the solution to realise productivity growth among hospitals in the KSA is technical progress. To achieve such technical progress (innovation), the requirement is investment in more or better machinery, equipment, and structures that will make it possible for hospitals to produce greater output [16, 32]. Other studies have found that productivity growth can be achieved by raising physician productivity, which is in turn dependent on the compensation mechanism [17, 36–39].

By comparison, public hospitals recorded a higher annual decline of TFP of 6.0% than private hospitals whose annual decline was 4.8%. In terms of sources, the deterioration in TFP in both public and private hospitals resulted from technical regress. Therefore, in general, improving productivity in hospitals may require the uptake of newer and better technologies through investments in more and better machinery, equipment, and structures.

A few limitations of the study are worth noting. The first limitation is that unavailability of data led to exclusion of private outpatient clinics for some regions. Their exclusion was aimed at reducing the heterogeneity of data to improve the accuracy of estimating productivity changes, as the scale and scope of services provided by these clinics are vastly different from those provided for inpatient hospital treatment. The second limitation is that although changes in TFP and its components were estimated, we did not further investigate the determinants of such changes for each region. Thirdly, since data are not available at the hospital level, we were not able to calculate regional scores of changes in productivity and its components. Future research could focus on these issues. For more effective interventions, the exact nature of investments and/or modifications in management practices needed to raise productivity must be determined for each concerned hospital or region.

## Conclusion

The results of this study show that TFP in the delivery of healthcare services has deteriorated in the KSA over the period 2006–2018 on account of technical regress. This suggests that there are productivity gains that are yet to be realised within the delivery of healthcare services in the KSA given the existing resource bundle. To realise these productivity gains, it is imperative to make appropriate investments. The specific interventions required need to be assessed for each region and each hospital.

## Data Availability

The datasets generated and/or analysed during the current study are available from the Ministry of Health website. Access to data can be gained through https://www.moh.gov.sa/en/Ministry/Statistics/book/Pages/default.aspx.
